# Supplementary material for: Systematic and meta-analysis of factors associated with preeclampsia and eclampsia in sub-Saharan Africa
Source: PLoS One. 2020 Aug 19;15(8):e0237600. doi: 10.1371/journal.pone.0237600 (PMC7437916; doi:10.1371/journal.pone.0237600)
Supplement: S1 File — (DOCX) [file pone.0237600.s002.docx]

**Supplementary Figures for - Systematic and Meta-analysis of Factors Associated with Preeclampsia and Eclampsia in sub-Saharan Africa**

Fig S1: PRISMA flow chart for the selection of studies on risk factors of preeclampsia and eclampsia in SSA countries

Full-text articles excluded with reason
(n =42)

Screening

Eligibility

Included

Studies included for systematic review and meta-analysis
(n = 51)

Full-text articles assessed for eligibility
(n =93)

Articles identified through database search
(n =6813 )

Records screened
(n = 120)

Articles after duplicates removed
(n = 4805)

Identification

Records excluded based on their title
(n =4,685)

Articles excluded based on their abstract (n=27)

Fig S2: The association between previous preeclampsia/eclampsia and risk of preeclampsia/eclampsia in SSA

Fig S3: The association between family history of preeclampsia and eclampsia women with preeclampsia/eclampsia in SSA

Fig S4: The association between women’s BMI and risk of preeclampsia and eclampsia in SSA

Fig S5: The association between having a pre-existing medical condition and the risk of preeclampsia and eclampsia in SSA countries

Fig S6: The association between anaemia during pregnancy and the risk of preeclampsia/eclampsia in SSA countries

Fig S7: The association between level of maternal education and risk of preeclampsia and eclampsia in SSA countries

Fig S8: The association between drinking alcohol and risk of preeclampsia and eclampsia in SSA countries

Fig S9: The association between ANC visit and risk of preeclampsia and eclampsia in SSA countries

Fig S10: The association between parity and risk of preeclampsia and eclampsia in SSA countries
