# Supplementary material for: Systematic and meta-analysis of factors associated with preeclampsia and eclampsia in sub-Saharan Africa
Source: PLoS One. 2020 Aug 19;15(8):e0237600. doi: 10.1371/journal.pone.0237600 (PMC7437916; doi:10.1371/journal.pone.0237600)
Supplement: S1 Table — (DOCX) [file pone.0237600.s003.docx]

| **S1 Table 1 : Quality Assessment for research article** | | | | | | | | | | | | | |
| --- | --- | --- | --- | --- | --- | --- | --- | --- | --- | --- | --- | --- | --- |
| **-S.No** | **Authors** | **Question 1** | **Question 2** | **Question 3** | **Question 4** | **Question 5** | **Question 6** | **Question 7** | **Question 8** | **Question 9** | **Question 10** | **Total(10)** | **Quality of paper** |
| **1** | **Allen Meeme**., et al.**/2016** | **Yes** | **Yes** | **Yes** | **Yes** | **Yes** | **Yes** | **Yes** | **No** | **Yes** | **Clear** | **8** | **High** |
| **2** | **Dorah Mrema**., et al.**/2018** | **Yes** | **Yes** | **Yes** | **Yes** | **Yes** | **Yes** | **Yes** | **Yes** | **Yes** | **Clear** | **9** | **High** |
| **3** | **Jocelynn T. Owusu** ., et al.**/2013** | **Yes** | **Yes** | **Yes** | **No** | **No** | **Yes** | **No** | **No** | **Yes** | **Clear** | **8** | **Medium** |
| **4** | **Helen C. Okoye** ., et al.**/2016** | **Yes** | **Yes** | **Yes** | **Yes** | **Yes** | **Yes** | **Yes** | **Yes** | **Yes** | **Clear** | **9** | **High** |
| **5** | **Yifru B and Gezahegn E /2015** | **Yes** | **Yes** | **Yes** | **Yes** | **Yes** | **Yes** | **Yes** | **Yes** | **Yes** | **Clear** | **9** | **High** |
| **6** | **Charlotte T. Nguefack** ., et al.**/ 2018** | **Yes** | **Yes** | **Yes** | **Yes** | **Yes** | **Yes** | **Yes** | **Yes** | **Yes** | **Clear** | **8** | **High** |
| **7** | **Teklit Grum** ., et al. **/2017** | **Yes** | **Yes** | **Yes** | **Yes** | **Yes** | **Yes** | **Yes** | **Yes** | **Yes** | **Clear** | **9** | **High** |
| **8** | **M. A. Ikpen**., et al.**/2012** | **Yes** | **Yes** | **Yes** | **Yes** | **Yes** | **Yes** | **Yes** | **No** | **Yes** | **unclear** | **7** | **Medium** |
| **9** | **Mulualem Endeshaw**., et al.**/2015** | **Yes** | **Yes** | **Yes** | **Yes** | **Yes** | **Yes** | **Yes** | **Yes** | **Yes** | **Clear** | **8** | **High** |
| **10** | Teklit Grum et al. (2018) |  |  |  |  |  |  |  |  |  |  |  |  |
| **11** | **Ugochukwu Vincent Okafor**., et al.**/2009** | **Yes** | **No** | **Yes** | **Yes** | **unclear** | **Yes** | **No** | **No** | **No** | **unclear** | **5** | **Low** |
| **12** | **SAOkogbenin**., et al.**/2010** | **Yes** | **No** | **Yes** | **Yes** | **Yes** | **Yes** | **Yes** | **Yes** | **No** | **Clear** | **8** | **High** |
| **13** | **K. MAHOMED** ., et al.**/2007** | **Yes** | **Yes** | **Yes** | **Yes** | **Yes** | **Yes** | **Yes** | **No** | **Yes** | **Clear** | **9** | **High** |
| **14** | **Gilles Guerrier**., et al.**/2013** | **Yes** | **No** | **Yes** | **Yes** | **Yes** | **Yes** | **Yes** | **Yes** | **Yes** | **unclear** | **7** | **Medium** |
| **15** | **Nadir A. Ahmed**., et al.**/2019** | **Yes** | **No** | **Yes** | **Yes** | **Yes** | **Yes** | **Yes** | **Yes** | **Yes** | **Clear** | **8** | **High** |
| **16** | **Leonard O Ajah**., et al. **/2016** | **Yes** | **Yes** | **Yes** | **Yes** | **Yes** | **Yes** | **Yes** | **Yes** | **Yes** | **Clear** | **9** | **High** |
| **17** | **Grazyna A. Stanczuk**., et al.**/2007** | **Yes** | **unclear** | **Yes** | **Yes** | **Yes** | **Yes** | **Yes** | **No** | **Yes** | **unclear** | **8** | **Medium** |
| **18** | **Dorothy J. Vanderjagt**., et al.**/2004** | **Yes** | **No** | **Yes** | **Yes** | **No** | **Yes** | **Yes** | **No** | **Yes** | **Clear** | **7** | **Medium** |
| **19** | **Jonah Musa**., et al.**/2018** | **Yes** | **Yes** | **Yes** | **Yes** | **Yes** | **Yes** | **Yes** | **Yes** | **Yes** | **Clear** | **9** | **High** |
| **20** | **Oladapo Olayemi**., et al.**/2010** | **Yes** | **Yes** | **Yes** | **Yes** | **Yes** | **Yes** | **Yes** | **Yes** | **Yes** | **unclear** | **8** | **High** |
| **21** | **Aleksandar Rajkovic**., et al.**/2000** | **No** | **No** | **Yes** | **Yes** | **Yes** | **Yes** | **Yes** | **Yes** | **No** | **Clear** | **7** | **Medium** |
| **22** | **Rosemary J Pegoraro**., et al. **/2004** | **Yes** | **Yes** | **Yes** | **Yes** | **Yes** | **Yes** | **Yes** | **Yes** | **Yes** | **Clear** | **10** | **High** |
| **23** | **Babatunde Salako**., et al.**/2004** | **Yes** | **Yes** | **Yes** | **Yes** | **Yes** | **Yes** | **Yes** | **Yes** | **Yes** | **Clear** | **8** | **High** |
| **24** | **Abdelmageed Elmugabil** ., et al.**/2016** | **Yes** | **Yes** | **Yes** | **Yes** | **unclear** | **Yes** | **Yes** | **No** | **Yes** | **unclear** | **7** | **Medium** |
| **25** | **Mulualem Endeshaw** ., et al.**/2016** | **Yes** | **No** | **Yes** | **Yes** | **Yes** | **Yes** | **Yes** | **Yes** | **Yes** | **Clear** | **8** | **High** |
| **26** | **Oluranti B. Familoni**., et al.**/2004** | **Yes** | **Yes** | **Yes** | **Yes** | **Yes** | **Yes** | **Yes** | **Yes** | **No** | **Clear** | **7** | **Medium** |
| **27** | **IshagAdam** ., et al.**/2013** | **Yes** | **Yes** | **Yes** | **Yes** | **Yes** | **Yes** | **Yes** | **Yes** | **Yes** | **Clear** | **8** | **High** |
| **28** | **Gizachew Assefa Tessema**., et al. **/2015** | **Yes** | **No** | **Yes** | **Yes** | **Yes** | **Yes** | **Yes** | **Yes** | **Yes** | **Clear** | **8** | **High** |
| **29** | **Jurgen Wacker**., et al.**/2000** | **Yes** | **Yes** | **Yes** | **Yes** | **Yes** | **Yes** | **Yes** | **Yes** | **Yes** | **unclear** | **8** | **High** |
| **30** | **Rose I. Anorlu**., et al.**/2005** | **Yes** | **Yes** | **Yes** | **Yes** | **Yes** | **Yes** | **Yes** | **Yes** | **Yes** | **Clear** | **8** | **High** |
| **31** | **Paul Kiondo**., et al.**/2012** | **Yes** | **Yes** | **Yes** | **Yes** | **Yes** | **Yes** | **Yes** | **Yes** | **Yes** | **unclear** | **8** | **Medium** |
| **32** | **António Bugalho**., et al.**/2001** | **Yes** | **No** | **Yes** | **Yes** | **Yes** | **Yes** | **Yes** | **Yes** | **Yes** | **unclear** | **8** | **Medium** |
| **33** | **Annelies Immink**., et al.**/2008** | **Yes** | **No** | **No** | **Yes** | **Yes** | **Yes** | **Yes** | **No** | **No** | **unclear** | **5** | **Low** |
| **34** | **AbdelAziem A Ali/2011** | **Yes** | **Yes** | **Yes** | **Yes** | **Yes** | **Yes** | **Yes** | **Yes** | **Yes** | **Clear** | **9** | **High** |
| **35** | **K. A. Frank/2004** | **Yes** | **Yes** | **Yes** | **Yes** | **Yes** | **Yes** | **Yes** | **Yes** | **Yes** | **Clear** | **9** | **High** |
| **36** | **Kathleen M Powis** ., et al.**/2013** | **Yes** | **Yes** | **Yes** | **Yes** | **Yes** | **Yes** | **Yes** | **Yes** | **Yes** | **Clear** | **10** | **High** |
| **37** | **Candice B. Roberts** ., et al.**/2004** | **Yes** | **Yes** | **Yes** | **Yes** | **Yes** | **Yes** | **Yes** | **Yes** | **Yes** | **Clear** | **8** | **High** |
| **38** | **VMS Kalumba** ., et al.**/2013** | **Yes** | **Yes** | **Yes** | **Yes** | **Yes** | **Yes** | **Yes** | **Yes** | **Yes** | **Clear** | **8** | **High** |

CASP Key questions

1. Question 1- Did the study address a clearly stated objective?
2. Question 2- Was the study accurately measured to minimise bias?
3. Question 3- Was the target population specified ?
4. Question 4- Was the study use appropriate study method?
5. Question 5- How precise the tools used to measure the results?
6. Question 6- was the response rate adequate?
7. Question 7- Do the results of the study fit with other available evidence ?
8. Question 8- Can the results be applied to the local population?
9. Question 9- Does the paper answered objective clearly?
10. Question `10- What are the implications of this study for practice?
