# Supplementary material for: Systematic and meta-analysis of factors associated with preeclampsia and eclampsia in sub-Saharan Africa
Source: PLoS One. 2020 Aug 19;15(8):e0237600. doi: 10.1371/journal.pone.0237600 (PMC7437916; doi:10.1371/journal.pone.0237600)
Supplement: Attachment — Submitted filename: Response to Reviewers.docx [file pone.0237600.s005.docx]

**Response to reviewers**

Reviewer #: Thank you once again for your constructive feedback and comments.

Reviewer #2: Excellent work! Thank for your corrections. Only minor edit recommended would be:
(line 460-463)
“Not only woman having previous preeclampsia/eclampsia have an increased risk of preeclampsia/eclampsia, but also having a family history of preeclampsia/eclampsia has positive association with present pregnancy.”

Response: Thank you for your constructive comment that will make the manuscript more readable. We have rewritten the sentence as per your guide and advice. (Line 444-445)

“Women with family histories of preeclampsia/eclampsia were noted to be at an increased risk for development of preeclampsia/eclampsia in the subsequent pregnancies.”
